# Seasonal Variations in Mood and Behavior in Romanian Postgraduate Students

**DOI:** 10.1100/tsw.2007.127

**Published:** 2007-06-12

**Authors:** Joseph J. Soriano, Constantin Ciupagea, Kelly J. Rohan, Dorin B. Neculai, Samina M. Yousufi, Alvaro Guzman, Teodor T. Postolache

**Affiliations:** ^1^ Mood and Anxiety Program, Department of Psychiatry, University of Maryland, 685 West Baltimore Street, MSTF Building Room 502, Baltimore, MD 21201, USA; ^2^ Romanian Centre for Economic Modeling, Bucharest, Sector 4, Radulescu-Motru Str. Nr. 14, Bucharest, Romania; ^3^ Psychology Department, University of Vermont, John Dewey Hall, 2 Colchester Avenue, Burlington, VT 05405, USA; ^4^ The Institute and Hospital “Professor Dr. Dorin Hociota”, Str. Mihai Cioranu Nr. 21 Bucharest, Romania; ^5^ Residency Training Program, District of Columbia Department of Mental Health and St. Elizabeths Hospital, 2700 Martin Luther King Avenue, Washington, D.C. 20032, USA

**Keywords:** seasonal affective disorder, summer type SAD, ethnic differences, standard of living impact, heat, environmental temperature, air conditioning, environmental light

## Abstract

To our knowledge, this paper is the first to estimate seasonality of mood in a predominantly Caucasian sample, living in areas with hot summers and a relative unavailability of air conditioning. As a summer pattern of seasonal depression was previously associated with a vulnerability to heat exposure, we hypothesized that those with access to air conditioners would have a lower rate of summer seasonal affective disorder (SAD) compared to those without air conditioning. A convenience sample of 476 Romanian postgraduate students completed the Seasonal Pattern Assessment Questionnaire (SPAQ), which was used to calculate a global seasonality score (GSS) and to estimate the rates of winter- and summer-type SAD. The ratio of summer- vs. winter-type SAD was compared using multinomial probability distribution tests. We also compared the ratio of summer SAD in individuals with vs. without air conditioners. Winter SAD and winter subsyndromal SAD (S-SAD) were significantly more prevalent than summer SAD and summer S-SAD. Those with access to air conditioners had a higher, rather than a lower, rate of summer SAD. Our results are consistent with prior studies that reported a lower prevalence of summer than winter SAD in Caucasian populations. Finding an increased rate of summer SAD in the minority of those with access to air conditioners was surprising and deserves replication.

### INTRODUCTION

Research on seasonality has focused primarily on winter depression and its relationship to short days. However, with global warming, a greater focus on human mental health vulnerability to heat and summer conditions, including increased risk for suicide, is beginning to emerge[1]. In his comprehensive review on weather and suicide, Deisenhammer described several studies that found a positive association between temperature and suicide, while a small number of studies did not[2]. Generally, an increase in suicide rates with increased temperature has been reported[3-9], although other reports conflict with this finding[10,11,12]. One would not be surprised by this relationship, as certain neurotransmitters of thermoregulation, such as norepinephrine, dopamine, and serotonin, are also key neurotransmitters of mood regulation. For instance, while exposure to sunlight is associated with increased serotonin turnover in the brain[13], higher ambient temperatures and relative humidity are associated with decreased availability of the serotonin precursor tryptophan[14].

Several studies reported an association between depression and ambient temperature. Shapira et al. examined 11 years of admissions data in a large, non-SAD (seasonal affective disorder) population of both bipolar depressive and unipolar patients, and discovered a positive correlation between admission rates and monthly temperatures in bipolar depressives, but not major depressive disorder patients[15]. Even in subtropical Taiwan with a fairly constant climate year-round, depressive episodes were seen to rise along with slight variations in temperature[16]. In the U.K., admissions rate for mania were monitored and found to be significantly higher in the summer with a trough in winter, and mean daily temperature was also found to be correlated with admissions[17].

If heat has adverse affective and behavioral effects in certain individuals, it is important to initially estimate and then demonstrate the degree of protection, if any, conferred by an air-conditioned microclimate.

Seasonality is defined as changes in mood, energy, sleep length, appetite, weight, and social activities across the seasons[18]. Rosenthal et al. (1984) first delineated the clinical features of fall/winter depression, called winter-type SAD[25]. SAD is described in DSM-IV as recurrent major depressive episodes during a particular time of year (e.g., in the fall or winter) with a full remission or a change to hypomania or mania at a particular time of the year (e.g., spring or summer). Subsyndromal SAD (S-SAD) is a milder form of SAD with similar symptoms, but less severe impairment[18].

Although winter-type SAD is more prevalent, a summer-type SAD that involves recurrent major depression in summer with remission in fall and winter has also been identified[19,20]. There are several distinctions between winter- and summer-type SAD. Whereas winter-type SAD is characterized by atypical depressive symptoms, such as anergia, oversleeping, overeating, carbohydrate craving, and weight gain, summer-type SAD has more typical depressive symptoms, such as agitation, insomnia, and weight loss[21]. Light deficiency, especially shortened photoperiods, is the hypothesized etiological mechanism behind winter-type SAD, and bright light treatment is the treatment of choice[22,23]. In contrast, summer-type SAD appears to be triggered by heat and humidity, and improves with temperature manipulations[21,24].

As another distinction, winter- and summer-type SAD are more severe and prolonged at higher and lower latitudes, respectively[24,25]. However, other studies have not found a correlation between latitude and seasonality, suggesting instead that genetic selection may have occurred in certain geographic areas or that there may be a ceiling effect in human response to short photoperiods[26,27,28,29]. In Caucasian samples, epidemiological research suggests that winter-type SAD is more prevalent than summer-type SAD[18,27,30,31,32,33,34].

In contrast to studies on Caucasians, Asian studies with Japanese civil servants[35], Filipino city workers[36], Thai residents[37], and Chinese students[38,39] have generally found a higher rate of summer-type than winter-type SAD. As the Thai and Filipino studies were performed at lower latitudes (i.e., warmer climates), the results may be attributed to increased heat. The Japanese and Chinese studies were performed at latitudes comparable to Montgomery County, Maryland in the U.S. where Kasper et al. found higher rates of winter- than summer-type SAD[18]; however, the great majority of Montgomery County residents have access to air conditioners in their homes as well as at work, whereas the availability of air conditioners is scarce in China.

This cross-sectional study is the first to estimate seasonality in Romanians. However, the major aim of the study is broader: To compare summer and winter SAD rates in Caucasians who are exposed to hot summers, with relatively minimal access to air conditioners. If the seasonal pattern in our sample is found to be similar to previous Caucasian studies, ethnicity likely explains the previously reported differences in seasonal patterns between Asians and Caucasians. On the other hand, if the seasonal pattern observed here appears similar to Asians, macro- and microenvironmental conditions most likely explain the differences between the previously reported differences in seasonal patterns between Asians and Caucasians.

We will test for the interactive effects of age, gender, period of living in similar climate conditions, awareness of SAD, and access to air conditioning systems on the degree of seasonality. Consistent with prior work, we expect greater seasonality among females than males[18,32,34,40,41,42,43] and among individuals who have previously heard about SAD[18], and a negative relationship between seasonality and age[18,27,28,32,34,44]. Finally, we will compare rates of summer SAD in students with vs. without air conditioners, hypothesizing that availability of air conditioners will be associated with lower rates of summer SAD.

### METHODS

Bucharest is the capital of Romania and is located at 45° northern latitude, which is similar to the location of Beijing, China (predominantly Asian population) where seasonality studies were previously performed[45]. The climate is moderately continental in both cities, specific to the inland regions of continents, with quite sharp differentiation of seasons in terms of humidity and temperature. The average annual temperature in Bucharest, reported for the latest 100 years (Romanian Statistical Yearbook, 2001), was 11°C (or 43°F), with an amplitude around 25°C (51°F) between monthly temperature averages (July vs. January). During the latest decade, the temperature pattern seemed to come even closer to the continental type of climate, as the winter-summer gap increased by 6.7°F (3.4°C). The average temperature during winter months fluctuates in the range of 25–37°F (–3.6 to 2.8°C), while during summer months, it stays within 76–82°F (22.3–25°C). There is also a large registered temperature gap between day and night, often higher than 25°F (12–13°C), which means that during night time, the average monthly temperature may go below 20°F (–6°C), with rather long periods of minimum temperatures during nights dropping below 12°F. Romania is a relatively poor country in comparison to European standards. The extreme poverty rate (measured according to subjective type of methods, thus taking the cost of living into account and considering the food poverty line calculated on the basis of a standard consumption basket) is close to 10% of the total population. It is also true that in the Bucharest metropolitan area, the poverty incidence is not as high as in the rest of the country, and is definitely not as high among the medical students or their households. As a consequence of the low standard of living, there is a certain lack of resources for the procurement of air conditioning systems and, therefore, the availability of such equipment in houses or in offices is recorded to be very low, although increasing. The overwhelming majority of the Romanian population (more than 99.5%) is Caucasian.

#### Participants

Participants were medical students at Bucharest Medical School, Romania (Universitatea de Medicina, Institutul de Medicina si Farmacie, Bucurest). We used the same study methodology performed among Chinese medical students in the Beijing area[39], in order to (a) evaluate the prevalence of SAD in medical students exposed to a similar climate and a relative paucity of air conditioners, but of Caucasian (rather than Asian) ethnicity and (b) to assess whether air conditioning use during the hot summers is associated with a lower rate of summer SAD.

#### Measures

Since its development, the Seasonal Pattern Assessment Questionnaire (SPAQ; [22]) has been widely used to assess degree of seasonality in studies with both clinical and normal populations[18,27,28,32,33,

34,42,44,45,46,47,48]. The SPAQ has also been used with college students[38,39,49,50,51,52], with a high degree of test-retest reliability[50]. The SPAQ contains questions about changes in mood and behavior across the seasons. Six items (i.e., mood, sleep, appetite, weight, energy, social activities) are rated on a 0 (no change) to 4 (extreme change) scale and are summed to compute a global seasonality score (GSS)[18]. An affirmative response to the question "If you experience changes with the seasons, do you feel that these are a problem for you?" is used to identify respondents who perceive seasonality as a problem. Having heard of SAD was considered an indicator of awareness of the disorder, measured as a binary variable (yes, no).

SPAQ-derived criteria for SAD are (1) GSS ≥ 11, (2) reporting that seasonal changes pose a problem, and (3) rating the problem severity as at least moderate[18]. S-SAD criteria on the SPAQ are (1) GSS = 9–10 or (2) GSS ≥ 11, and indicating no problems or only a mild problem with seasonal changes[41]. In response to the SPAQ question "At what time of year do you feel worst?", a response of December, January, and/or February receives the winter-type specifier to a SAD or S-SAD diagnosis. Feeling worst during June, July, and/or August warrants the summer-type specifier.

### Procedure

The SPAQ was administered to students in a classroom/laboratory setting. The participants, who were all medical students, gave their consent for answering the SPAQ questions following the full explanation of the study to be performed.

### Statistical Analyses

To examine the impact of age, gender, period of living under similar conditions (duration of residence in the study area), access to air conditioning systems, and general awareness of SAD on seasonality, we performed an analysis of variance (ANOVA) with GSS as the dependent variable and duration of residence in the area, gender, access to air conditioning, and awareness as independent factors. The possible effect of age on the expected results was examined in an ANCOVA procedure. Because these relations have not been tested in prior studies, we used two-tailed tests in all analyses. We descriptively compared rates of winter- and summer-type SAD, total SAD, and mood pattern between female and male students. Chi-Square analyses were conducted to test the hypothesized pattern of gender differences in winter- and summer-type diagnoses. Rates of summer and winter SAD were compared using polynomial tests. The rate of summer SAD was compared in those with vs. without air conditioners using chi-square tests. For each analysis, we used all available data (i.e., data from all participants who reported on the variables of interest for each analysis). Questionnaires with incomplete data were not discarded, with all available information used for analysis.

## RESULTS

### Characteristics of the Sample

A convenience sample of 476 postgraduate students (out of 488 invited to participate) completed the SPAQ[53], which was used to calculate a GSS and to estimate the rates of winter- and summer-type SAD. The resulting student sample (N = 476) contained 326 female students (68.49%) and 150 male students (31.51%); 472 students (99.1%) gave information concerning their age. The average age of the entire medical students sample was 23.2 years (SD = 2.9), the average age of male students was 23.8 years (SD = 3.7, range = 19–49), and the average age of female students was 22.95 years (SD = 2.44, range = 19–35). The average duration of residence in the study area was 13.26 years (SD = 9.36) for the entire sample, 12.85 years (SD = 9.1) for female students, and 14.14 years (SD = 9.9) for male students. Regarding awareness of SAD, out of the 474 students who answered this question, 311 (65.6%) were aware of the existence of the disorder. The majority of female (67.6%) and male (60.7%) students reported having heard about SAD. Only 8.8% of the total sample (42 out of 476) reported availability of air conditioning devices at their offices/places of work/places of learning, and even fewer (N = 25; 5.3% of the total sample) reported having access to such devices in their homes. The overall number of students accessing air conditioning systems at home and/or at their work place was 61 (corresponding to 12.8% of the total sample). The majority of participants were Caucasians (473 out of 476 respondents; 99.3%).

### Seasonality and Age, Gender, Access to Air Conditioning Systems, and Awareness of SAD

Table 1 presents GSS by gender, access to an air conditioning system, and awareness of SAD. The mean GSS for the entire sample was 7.82 with a standard deviation of 4.0. The GSS was not significantly related to awareness of SAD or access to air conditioning systems. Nonetheless, the analysis shows a strong relationship between gender and GSS, with a higher GSS in females. Female students had a significantly higher mean GSS (M = 8.24, SD = 3.85) than male students (M = 6.92, SD = 3.66). In contrast to other previous studies in the general population, GSS did not correlate negatively with age in our sample, r = 0.037, $p$ = 0.493.

**TABLE 1**
**GSS by awareness of SAD, Access to Air Conditioning Systems, and Gender**

| | Access to Air Conditioning Systems (Either Home or Office) | | | Total Sample | | |
|---|---|---|---|---|---|---|
| | M | SEM | No. | M | SEM | No. |
| Total sample | | | | | | |
| Aware of SAD | 7.025 | 0.544 | 40 | 7.897 | 0.515 | 311 |
| Unaware of SAD | 8.850 | 0.448 | 20 | 7.685 | 0.506 | 163 |
| Females | | | | | | |
| Aware of SAD | 7.148 | 0.498 | 27 | 8.386 | 0.487 | 220 |
| Unaware of SAD | 8.667 | 0.616 | 9 | 7.952 | 0.524 | 104 |
| Males | | | | | | |
| Aware of SAD | 6.769 | 0.658 | 13 | 6.714 | 0.565 | 91 |
| Unaware of SAD | 9.000 | 0.294 | 11 | 7.237 | 0.453 | 59 |

### Rate of SAD and Seasonality by Gender, Awareness, and Accessibility to Air Conditioning

Rate estimates for winter- and summer-type SAD, S-SAD, and total SAD (i.e., a category combining SAD and S-SAD) by gender, awareness, and accessibility to air conditioning are presented in Table 2. A total of 18 students (3.78%) met criteria for winter SAD and an additional 35 (7.35%) met criteria for winter S-SAD. Fewer students reported summer SAD (N = 6, 1.26%) and summer S-SAD (N = 5, 1.05%). A significantly larger proportion of students met criteria for winter SAD than summer SAD (3.78 vs. 1.26%; Z = 2.4651, $p$ = 0.036), somehow in contradiction with the hypothesized similarity to the

**TABLE 2**
**Prevalence Estimates for Winter- and Summer-type SAD and Total SAD, by Gender, Awareness, and Accessibility to Air Conditioning Systems**

| | Total Sample (N = 476) | Aware of (N = 311) | Access to Air Conditioning (N = 61) |
|---|---|---|---|
| Winter-type SAD, No. (%) | 18/476 (3.78%) | 8/311 (2.57%) | 3/61 (4.92%) |
| Females, No. (%) | 17/326 (5.21%) | 8/220 (3.64%) | 2/40 (5.00%) |
| Males, No. (%) | 1/150 (0.67%) | 0/91 (0.00%) | 1/20 (5.00%) |
| Summer-type SAD, No. (%) | 6/476 (1.26%) | 4/311 (1.28%) | 4/61 (6.56%) |
| Females, No. (%) | 2/326 (0.61%) | 2/220 (0.91%) | 2/40 (5.00%) |
| Males, No. (%) | 4/150 (2.67%) | 2/91 (2.20%) | 2/20 (10.00%) |
| Winter-Total-SAD, No. (%) | 53/476 (11.13%) | 34/311 (10.93%) | 6/61 (9.84%) |
| Females, No. (%) | 40/326 (12.27 %) | 27/220 (12.27%) | 3/40 (7.50%) |
| Males, No. (%) | 13/150 (8.67%) | 7/91 (7.69%) | 3/20 (15.00%) |
| Summer-Total-SAD, No. (%) | 11/476 (2.31%) | 8/311 (2.57%) | 5/61 (8.20%) |
| Females, No. (%) | 4/326 (1.23%) | 3/220 (1.36%) | 2/40 (5.00%) |
| Males, No. (%) | 7/150 (4.67%) | 5/91 (5.49%) | 3/20 (15.00%) |

Chinese medical student study[39]. For total SAD (cumulative category for SAD and S-SAD cases), the proportion of total winter SAD (11.3%) was higher than the proportion of total summer SAD (2.31%), Z = 5.409, $p = 0.006$.

### Gender Effects

Female students showed a significantly higher rate of winter SAD than summer SAD, and a higher rate of winter SAD than male students. Males had a tendency towards a greater rate of summer SAD than winter SAD, and higher rate of summer SAD than female students, but the sample size was too small to come to any conclusions (see Tables 2 and 3).

**TABLE 3**
**Prevalence of Winter- vs. Summer-Type S-SAD and Total SAD by Gender**

| | Winter Pattern (%) | Summer Pattern (%) | z-Test | *p* |
|---|---|---|---|---|
| Total sample | | | | |
| S-SAD | 3.78 | 1.26 | 2.465 | 0.038 |
| Total SAD | 11.13 | 2.31 | 5.409 | 0.006 |
| Female students | | | | |
| S-SAD | 5.21 | 0.61 | 3.506 | 0.020 |
| Total SAD | 12.27 | 1.23 | 5.690 | 0.005 |
| Male students | | | | |
| S-SAD | 0.67 | 2.67 | –1.34 | NS |
| Total SAD | 8.67 | 4.67 | 1.34 | NS |

### Availability of Air Conditioners

Of the total 476 students, 42 (8.8%) reported availability of air conditioning devices at their work/learning place, 25 (5.3%) reported access to such devices in their homes, and 6 (1.2%) had access to air conditioning at both their work/learning place and home.

Contrary to our hypothesis, those with an air conditioner had a higher, not a lower, rate of summer total SAD (Fisher exact test two tail $p < 0.007$). The rate of winter total SAD did not differ in those with vs. those without air conditioners (Table 4).

**TABLE 4**
**Winter and Summer Total SAD and Accessibility to Air Conditioning**

| | No Access (N = 415) | Access (N = 61) | *p* |
|---|---|---|---|
| Winter Total SAD, No. (%) | 47/415 (11.56%) | 5/61 (8.19%) | 0.730 |
| Summer Total SAD, No. (%) | 6/415 (1.44%) | 5/61 (8.20%) | 0.007 |

### DISCUSSION

This is the first time that seasonality of mood was evaluated in a Caucasian sample with little access to air conditioners during the hot summers. Despite Romanian hot summers, winter SAD was more prevalent than summer SAD in this medical student sample, a finding consistent with previous studies in climates with milder summers, at coastal locations, or in places where air conditioning is more widely available. Thus, it is unlikely that climate and microclimate is responsible for the observed differences in seasonality patterns between Asian and Caucasian samples. Instead of macro- or microclimatic factors, it is likely that ethnicity, with its multiple genetic and cultural implications, explains why Asians are relatively resilient to winter SAD and more vulnerable to summer SAD.

Contrary to our expectation, students with access to air conditioners had a higher rate of summer-type SAD than those without access to air conditioners. This could be due to a self-selection of those who are more vulnerable to heat who invest in air conditioners (e.g., already vulnerable to SAD). Alternatively, other hidden variables related to physical, chemical, or biological environments of socioeconomic factors could play a role.

A plausible explanation could be that an acclimatized microenvironment would make individuals spend more time indoors during the summer, thus receiving less light and becoming more depressed. This would be unlikely, however, because participants with air conditioners had lower (rather than higher) rates of winter SAD than participants without air conditioners, suggesting that participants with air conditioners did not have a vulnerability to depression related to light deprivation. Similarly, as is common in winter in colder climates, spending more time indoors may expose individuals to factors that may increase inflammation in the upper airway (i.e., viruses, bacteria, particulate matter, and allergens), potentially which may result in worsening of mood, depression, and anxiety[54,55]. An alternative explanation may be that spending more time indoors than outdoors during summer may reduce exercise and social activities specific to summer, resulting in mood worsening.

The reported gender effect (a greater winter SAD rate in *females*, but a higher summer SAD rate in *males)*, if replicated in another country with hot summers and reduced availability of air conditioned microclimates, may reflect a differential vulnerability in females to light and day length and in males to heat. Changes in meteorological conditions (e.g., temperature, luminosity, humidity) may represent an external environmental cue that could potentially precipitate changes in brain neuronal activity in predisposed individuals, resulting in mood and behavioral changes.

### Limitations of the Paper

As a self-report tool, the SPAQ generally tends to overestimate SAD in comparison to clinical diagnostic interviews[56]. Mersch et al. state that the SPAQ is not adequate to be used in the clinical diagnosis of SAD, but only as a screening tool[57]. The Romanian version of the SPAQ is not yet formally validated. Medical students likely do not represent the Romanian population as a whole. Certainly, outdoor workers would be more exposed to heat during summer. The living standards in Romania are rapidly changing, with the expectation that more and more individuals will have access to air conditioners during summer.

In conclusion, in contrast to Asian studies, even where air conditioners are not available and summers are hot, summer SAD is less frequent than winter SAD in our predominately Caucasian Romanian subjects. The presence of air conditioners was associated with a greater (not lower, as hypothesized) rate of summer SAD, a finding that we do not yet fully understand. Females appeared relatively less prone to summer SAD than males. Considering the highly anticipated warming of the planet, additional research on behavioral effects of heat and its interaction with gender, ethnicity, and microclimates is highly justified.

## References

1. Preti, A., Lentini, G., and Maugeri, M. (2007) Global warming possibly linked to an enhanced risk of suicide: data from Italy, 1974–2003. *J. Affect. Disord*., in press.
2. Deisenhammer, E.A. (2003) Weather and suicide: the present state of knowledge on the association of meteorological factors with suicidal behaviour. *Acta Psychiatr. Scand*. **108**, 402–409.
3. Breuer, H.W., Breuer, J., and Fischbach-Breuer, B.R. (1986) Social, toxicological and meteorological data on suicide attempts. *Eur. Arch. Psychiatry Neurol. Sci*. **235,** 367–370.
4. Souetre, E., Wehr, T.A., Douillet, P., and Darcourt, G. (1990) Influence of environmental factors on suicidal behavior. *Psychiatry Res.* **32,** 253–263.
5. Maes, M., De Meyer, F., Thompson, P., Peeters, D., and Cosyns, P. (1994) Synchronized annual rhythms in violent suicide rate, ambient temperature and the light-dark span. *Acta Psychiatr. Scand.* **90,** 391–396.
6. Salib, E. and Gray, N. (1997) Weather conditions and fatal self-harm in North Cheshire 1989–1993. *Br. J. Psychiatry* **171,** 473–477.
7. Preti, A. and Miotto, P. (2000) Influence of method on seasonal distribution of attempted suicides in Italy. *Neuropsychobiology* **41**, 62–72.
8. Lee, H.C., Lin, H.C., Tsai, S.Y., Li, C.Y., Chen, C.C., and Huang, C.C. (2006) Suicide rates and the association with climate: a population-based study. *J. Affect. Disord*. **92,** 221–226.
9. Linkowski, P., Martin, F., and De Maertelaer, V. (1992) Effect of some climatic factors on violent and non-violent suicides in Belgium. *J. Affect. Disord*. **25**, 161–166.
10. Tietjen, G.H. and Kripke, D.F. (1994) Suicides in California (1968–1977): absence of seasonality in Los Angeles and Sacramento counties. *Psychiatry Res*. **53**, 161–172.
11. Dixon, P.G., McDonald, A.N., Scheitlin, K.N., Stapleton, J.E., Allen, J.S., Carter, W.M., Holley, M.R., Inman, D.D., and Roberts, J.B. (2007) Effects of temperature variation on suicide in five U.S. counties, 1991–2001. *Int. J. Biometeorol*. **51(5),** 395–403.
12. Ajdacic-Gross, V., Lauber, C., Sansossio, R., Bopp, M., Eich, D., Gostynski, M., Gutzwiller, F., and Rossler, W. (2007) Seasonal associations between weather conditions and suicide--evidence against a classic hypothesis. *Am. J. Epidemiol*. **165,** 561–569.
13. Lambert, G.W., Reid, C., Kaye, D.M., Jennings, G.L., and Esler, M.D. (2002) Effect of sunlight and season on serotonin turnover in the brain. *Lancet* **360,** 1840–1842.
14. Maes, M., Scharpe, S., Verkerk, R., D'Hondt, P., Peeters, D., Cosyns, P., Thompson, P., De Meyer, F., Wauters, A., and Neels, H. (1995) Seasonal variation in plasma L-tryptophan availability in healthy volunteers. Relationships to violent suicide occurrence. *Arch. Gen. Psychiatry* **52**, 937–946.
15. Shapira, A., Shiloh, R., Potchter, O., Hermesh, H., Popper, M., and Weizman, A. (2004) Admission rates of bipolar depressed patients increase during spring/summer and correlate with maximal environmental temperature. *Bipolar Disord*. **6**, 90–93.
16. Lee, H.C., Tsai, S.Y., and Lin, H.C. (2007) Seasonal variations in bipolar disorder admissions and the association with climate: a population-based study. *J. Affect. Disord*. **97**, 61–69.
17. Myers, D.H. and Davies, P. (1978) The seasonal incidence of mania and its relationship to climatic variables. *Psychol. Med*. **8,** 433–440.
18. Kasper, S., Wehr, T.A., Bartko, J.J., Gaist, P.A., and Rosenthal, N.E. (1989) Epidemiological findings of seasonal changes in mood and behavior. A telephone survey of Montgomery County, Maryland. *Arch. Gen. Psychiatry* **46,** 823–833.

19. Boyce, P. and Parker, G. (1988) Seasonal affective disorder in the southern hemisphere. *Am. J. Psychiatry* **145,** 96–99.
20. Wehr, T.A. (1989) Seasonal affective disorder. A historical overview. In *Seasonal Affective Disorder and Phototherapy*. Rosenthal, N.E. and Blehar, M.C., Eds. Guilford, New York.
21. Wehr, T.A., Giesen, H.A., Schulz, P.M., Anderson, J.L., Joseph-Vanderpool, J.R., Kelly, K., Kasper, S., and Rosenthal, N.E. (1991) Contrasts between symptoms of summer depression and winter depression. *J. Affect. Disord.* **23,** 173–183.
22. Rosenthal, N.E., Genhart, M.J., Sack, D.A., Skwerer, R.J., and Wehr, T.A. (1987) Seasonal affective disorder: relevance for treatment and research of bulimia. In *The Psychobiology of Bulimia*. Hudson, J.I. and Pope, H.G., Eds. American Psychiatric Press, Washington, D.C.
23. Terman, M., Terman, J.S., and Ross, D.C. (1998) A controlled trial of timed bright light and negative air ionization for treatment of winter depression. *Arch. Gen. Psychiatry* **55,** 875–882.
24. Wehr, T.A., Sack, D.A., and Rosenthal, N.E. (1987) Seasonal affective disorder with summer depression and winter hypomania. *Am. J. Psychiatry* **144,** 1602–1603.
25. Rosenthal, N.E., Sack, D.A., Gillin, J.C., Lewy, A.J., Goodwin, F.K., Davenport, Y., Mueller, P.S., Newsome, D.A., and Wehr, T.A. (1984) Seasonal affective disorder. A description of the syndrome and preliminary findings with light therapy. *Arch. Gen. Psychiatry* **41,** 72–80.
26. Partonen, T., Partinen, M., and Lonnqvist, J. (1993) Frequencies of seasonal major depressive symptoms at high latitudes. *Eur. Arch. Psychiatry Clin. Neurosci.* **243,** 189–192.
27. Magnusson, A. and Axelsson, J. (1993) The prevalence of seasonal affective disorder is low among descendants of Icelandic emigrants in Canada. *Arch. Gen. Psychiatry* **50,** 947–951.
28. Broman, J.E. and Hetta, J. (1998) Prevalence of Seasonal Affective Disorder and Related Symptoms at Two Latitudes in Sweden. Tenth Annual Meeting on Light Treatment and Biological Rhythms. 228. Amelia Island, FL. Society for Light Treatment and Biological Rhythms, Chincoteague, VA.
29. Magnusson, A. (2000) An overview of epidemiological studies on seasonal affective disorder. *Acta Psychiatr. Scand.* **101,** 176–184.
30. Lingiaerde, O., Bratlid, T., Hansen, T., and Gotestam, K.G. (1986) Seasonal Affective Disorder and Midwinter Insomnia in the Far North: Studies on Two Related Chronobiological Disorders in Norway. 15th Collegium International Neuro-Psychopharmacologicum.
31. Potkin, S.G., Zetin, M., Stamenkovic, V., Kripke, D., and Bunney, W.E. (1986) Seasonal Affective Disorder: Prevalence varies with Latitude and Climate. 15th Collegium International Neuro-Psychopharmacologicum.
32. Rosen, L.N., Targum, S.D., Terman, M., Bryant, M.J., Hoffman, H., Kasper, S.F., Hamovit, J.R., Docherty, J.P., Welch, B., and Rosenthal, N.E. (1990) Prevalence of seasonal affective disorder at four latitudes. *Psychiatry Res.* **31,** 131–144.
33. Rosen, L.N. and Rosenthal, N.E. (1991) Seasonal variations in mood and behavior in the general population: a factor-analytic approach. *Psychiatry Res.* **38,** 271–283.
34. Booker, J.M. and Hellekson, C.J. (1992) Prevalence of seasonal affective disorder in Alaska. *Am. J. Psychiatry* **149,** 1176–1182.
35. Ozaki, N., Ono, Y., Ito, A., and Rosenthal, N.E. (1995) Prevalence of seasonal difficulties in mood and behavior among Japanese civil servants. *Am. J. Psychiatry* **152,** 1225–1227.
36. Ito, A., Ichihara, M., Hisanaga, N., Ono, Y., Kayukawa, Y., Ohta, T., Okada, T., and Ozaki, N. (1992) Prevalence of seasonal mood changes in low latitude area: Seasonal Pattern Assessment Questionnaire score of Quezon City workers. *Jpn. J. Psychiatry Neurol.* **46,** 249.
37. Srisurapanont, M. and Intaprasert, S. (1999) Seasonal variations in mood and behaviour: epidemiological findings in the north tropics. *J. Affect. Disord.* **54,** 97–99.
38. Han, L., Wang, K., Cheng, Y., Du, Z., Rosenthal, N.E., and Primeau, F. (2000) Summer and winter patterns of seasonality in Chinese college students: a replication. *Compr. Psychiatry* **41,** 57–62.
39. Han, L., Wang, K., Du, Z., Cheng, Y., Simons, J.S., and Rosenthal, N.E. (2000) Seasonal variations in mood and behavior among Chinese medical students. *Am. J. Psychiatry* **157,** 133–135.
40. Booker, J.M., Hellekson, C.J., Putilov, A.A., and Danilenko, K.V. (1991) Seasonal depression and sleep disturbances in Alaska and Siberia: a pilot study. *Arctic Med. Res.* **Suppl**, 281–284.
41. Magnusson, A. and Stefansson, J.G. (1993) Prevalence of seasonal affective disorder in Iceland. *Arch. Gen. Psychiatry* **50,** 941–946.
42. Levine, M.E. (1995) Seasonal symptoms in the sub-Arctic. *Mil. Med.* **160,** 110–114.
43. Mersch, P.P., Middendorp, H.M., Bouhuys, A.L., Beersma, D.G., and van den Hoofdakker, R.H. (1999) The prevalence of seasonal affective disorder in The Netherlands: a prospective and retrospective study of seasonal mood variation in the general population. *Biol. Psychiatry* **45,** 1013–1022.
44. Dam, H., Jakobsen, K., and Mellerup, E. (1998) Prevalence of winter depression in Denmark. *Acta Psychiatr. Scand.* **97,** 1–4.
45. Muscettola, G., Barbato, G., Ficca, G., Beatrice, M., Puca, M., Aguglia, E., and Amati, A. (1995) Seasonality of mood in Italy: role of latitude and sociocultural factors. *J. Affect. Disord.* **33**, 135–139.

46. Wirz-Justice, A., Krauchi, K., Graw, P., Schulman, J., and Wirz, H. (1992) Seasonality in Switzerland: An Epidemiological Survey. Fourth Annual Meeting on Light Treatment and Biological Rhythms. 33. Bethesda, MD. Society for Light Treatment and Biological Rhythms, Chincoteague, VA.
47. Hagfors, C., Thorel, L., and Amed, M. (1995) Seasonality in Finland and Sweden. An Epidemiologic Study. Preliminary Results. Seventh Annual Meeting on Light Treatment and Biological Rhythms. 51. Frankfurt, Germany. Society for Light Treatment and Biological Rhythms, Chincoteague, VA.
48. Hegde, A.L. and Woodson, H. (1996) Prevalence of seasonal changes in mood and behavior during the winter months in central Texas. *Psychiatry Res*. **62,** 265–271.
49. Low, K.G. and Feissner, J.M. (1998) Seasonal affective disorder in college students: prevalence and latitude. *J. Am. Coll. Health* **47**, 135–137.
50. Rohan, K.J. and Sigmon, S.T. (2000) Seasonal mood patterns in a northeastern college sample. *J. Affect. Disord*. **59**, 85–96.
51. Agumadu, C.O., Yousufi, S.M., Malik, I.S., Nguyen, M.C., Jackson, M.A., Soleymani, K., Thrower, C.M., Peterman, M.J., Walters, G.W., Niemtzoff, M.J., Bartko, J.J., and Postolache, T.T. (2004) Seasonal variation in mood in African American college students in the Washington, D.C., metropolitan area. *Am. J. Psychiatry* **161**, 1084–9.
52. Guzman, A., Rohan, K.J., Yousufi, S.M., Jackson, M.A., Nguyen, M.T., Soriano, J.J., and Postolache, T.T. (2007). Mood sensitivity to seasonal changes in African college students living in the greater Washington D.C. metropolitan area. *TheScientificWorldJOURNAL* **7**, 584-591.
53. Rosenthal, N.E., Bradt, G.H., and Wehr, T.A. (1984) Seasonal Pattern Assessment Questionnaire (SPAQ). National Institute of Mental Health, Bethesda, MD.
54. Postolache, T.T., Komarow, H.D., Stiller, J.W., and Tonelli, L.H. (2005) Allergy, depression, and suicide. *Direct. Psychiatry* **25,** 59–69.
55. Tonelli, L.H. and Postolache, T.T. (2005) Tumor necrosis factor alpha, interleukin-1 beta, interleukin-6 and major histocompatibility complex molecules in the normal brain and after peripheral immune challenge. *Neurol. Res*. **27,** 679–684.
56. Blazer, D.G., Kessler, R.C., and Swartz, M.S. (1998) Epidemiology of recurrent major and minor depression with a seasonal pattern. The National Comorbidity Survey. *Br. J. Psychiatry* **172,** 164–167.
57. Mersch, P.P., Vastenburg, N.C., Meesters, Y., Bouhuys, A.L., Beersma, D.G., van den Hoofdakker, R.H., and den Boer, J.A. (2004) The reliability and validity of the Seasonal Pattern Assessment Questionnaire: a comparison between patient groups. *J. Affect. Disord*. **80,** 209–219.
